# Cytokine data obtained from synovial stromal cells of patients with rheumatoid arthritis or osteoarthritis

**DOI:** 10.1016/j.dib.2017.04.041

**Published:** 2017-04-29

**Authors:** Johanna A. Huhtakangas, Johanna Veijola, Sanna Turunen, Anna Karjalainen, Maarit Valkealahti, Tomi Nousiainen, Susanna Yli-Luukko, Olli Vuolteenaho, Petri Lehenkari

**Affiliations:** a Cancer and Translational Medicine Research Unit, Medical Research Center Oulu, Oulu University Hospital and University of Oulu, Oulu, Finland; b Rheumatology Unit, Department of Medicine, Oulu University Hospital and University of Oulu, Oulu, Finland; c Biochemistry and Molecular Medicine Research Unit, Medical Research Center Oulu, Oulu University, Oulu, Finland; d Division of Operative Care, Oulu University Hospital and University of Oulu, Oulu, Finland; e Biomedicine Unit, Department of Physiology, Medical Research Center Oulu, Oulu University, Oulu, Finland

**Keywords:** Cytokines, Rheumatoid arthritis, Osteoarthritis

## Abstract

In this article, we share the raw cytokine data obtained from basal and stimulated synovial stromal cells cultured from patients with rheumatoid arthritis or osteoarthritis. This data article is related to the research article entitled “1,25D_3_ and calcipotriol, its hypocalcemic analog, exert a long-lasting anti-inflammatory and anti-proliferative effect in synoviocytes cultured from patients with rheumatoid arthritis and osteoarthritis (1). Cytokine levels were analyzed by a magnetic bead–based multiplex assay (a panel of 27 important cytokines) in two separate sets of experiments. The first was conducted with IL-1β and 1,25(OH)_2_D_3_ and the other with TNFα, calcipotriol, i.e. the hypocalcemic analog 1,25(OH)_2_D_3_, and dexamethasone. The raw data of this article display the individual variation in basal secretion of cytokines and in their response to different stimuli.

**Specifications table**

**Table:** 

Subject area	Translational science
More specific subject area	Inflammation in the synovium of rheumatoid arthritis and osteoarthritis patients
Type of data	Tables, text
How data was acquired	Cytokines were measured by the Luminex MagPix Instrument and Luminex xPotent Software and Bio-Rad Bio-Plex Pro Human Cytokine Grp I Panel (27-plex) PGE_2_ data is produced by ELISA (Thermo Scientific, USA)
Data format	raw cytokine data
Experimental factors	Basal and exposed samples from the cell culture media were collected and analyzed for cytokines.
Experimental features	Cytokine levels were analyzed by a magnetic bead–based multiplex assay (a panel of 27 important cytokines) after a 48-h treatment with different stimuli
Data source location	Oulu, Finland, 65°01′N, 025°28′E
Data accessibility	Data is with this article

**Value of the data**

• The raw cytokine data in this article reveal the natural variation of cytokine levels in cultured synovial stromal cells obtained from patients with rheumatoid arthritis or osteoarthritis.
• This data shows absolute cytokine levels in baseline and stimulated states (stimulated with TNF-α or IL-1β) and when treated with anti-inflammatory factors [1,25(OH)_2_D_3_, calcipotriol or dexamethasone].
• By examining the individual responses to different stimuli, it is possible to evaluate the individual and overall significance of different cytokines in the synovial pathology of rheumatoid arthritis and osteoarthritis. This kind of data can help to identify the most important cytokines for further analysis by other research groups.

## 1 Data

See Table 1, Table 2.

**Table 1:** Basal and stimulated levels of cytokines (pg/ml) after a 48-h exposure with IL-1β (10 ng/ml) and 1,25 (OH)_2_D_3_ (10 nM). The apparent level of IL-1β was mostly attributable to the added IL-1β. Data comprises cell cultures from four patients with osteoarthritis (OA) and four with rheumatoid arthritis (RA). Cytokines have been analyzed in two separate runs which explains the slight difference in the lower detection limit. The final data is presented in Fig. 8 of the research article [1].

**Cytokine**	**Patient**	**IL-1β**	**IL-1β+1.25 (OH)**_**2**_**D**_**3**_
MIP-1β	OA OA OA OA RA RA RA RA	27.5 136.7 44.2 25.3 33.21 48.6 74.82 <14.7	20.7 114.21 21.1 16.3 28.61 42 92.9 <14.7
IL-6	OA OA OA OA RA RA RA RA	>38026 >100917 >100917 >100917 >100917 >38026	15,859 30,910 16,158 39,876 78,084 27,060
IFN-γ	OA OA OA OA RA RA RA RA	65.1 83.5 103.7 104.6 71.5 73.4 114.9 94.1	43.1 76.1 72.5 77.2 56.1 70.3 109.3 77.8
IL-1ra	OA OA OA OA RA RA RA RA	147.1 159.0 196.1 200.1 173.0 177.5 216.0 182.5	93.1 <88.6 137.1 144.9 138.8 171.6 213.1 156.9
IL-5	OA OA OA OA RA RA RA RA	<24.73 <24.73 <24.73 <24.73 <24.73 <24.73 <24.73 <24.73	<24.73 <24.73 <24.73 <24.73 <24.73 <24.73 <24.73 <24.73
GM-CSF	OA OA OA OA RA RA RA RA	88.95 106.3 129.8 197.2 95.8 93.2 119.9 124.7	71.8 82.9 85.2 95.7 90.1 78.3 106.3 91.4
TNF-α	OA OA OA OA RA RA RA RA	<79.83 <79.83 <79.83 <79.83 <79.83 <79.83 <79.83 <79.83	<79.83 <79.83 <79.83 <79.83 148.59 <79.83 <79.83 <79.83
RANTES	OA OA OA OA RA RA RA RA	167.4 2716.0 476.0 223.5 489.4 1095 >1267 256.5	135.4 2897.0 336.6 187.5 493.9 927.3 1267 205.0
IL-2	OA OA OA OA RA RA RA RA	<13.52 14.51 15.21 16.24 <13.52 <13.52 15.57 15.06	<13.52 13.76 <14.83 <14.83 <13.52 <13.52 <14.83 <14.83
IL-1β	OA OA OA OA RA RA RA RA	98.0 76.5 95.4 96.8 77.8 85.3 96.3 97.8	71.4 62.9 87.1 108.4 70.5 74.7 83.8 102.4
Eotaxin	OA OA OA OA RA RA RA RA	<25.51 <25.51 <27.16 <27.16 <25.51 <25.51 <27.16 <27.16	<25.51 <25.51 <27.16 <27.16 <25.51 <25.51 <27.16 <27.16
Basic FGF	OA OA OA OA RA RA RA RA	25.3 34.3 36.3 33.3 42.3 28.8 48.5 34.3	22.5 36.9 29.2 28.8 26.6 28.4 40.5 27.3
VEGF	OA OA OA OA RA RA RA RA	477.7 1013.0 496.4 343.8 818.2 1060.0 1054.0 843.2	282.5 906.3 317.9 299.6 562.6 1032.0 1136.0 688.5
PDGF-BB	OA OA OA OA RA RA RA RA	<29.30 <29.30 <28.16 <28.16 <29.30 <29.30 <28.16 <28.16	<29.30 <29.30 <28.16 <28.16 <29.30 <29.30 <28.16 <28.16
IP-10	OA OA OA OA RA RA RA RA	96.7 704.9 <32.23 <32.23 158.9 264.8 <32.23 <32.23	88.1 638.0 <32.23 <32.23 286.6 234.6 <32.23 <32.23
IL-13	OA OA OA OA RA RA RA RA	<28.57 <28.57 <31.09 <31.09 <28.57 <28.57 <31.09 <31.09	<28.57 <28.57 <31.09 <31.09 <28.57 <28.57 <31.09 <31.09
IL-4	OA OA OA OA RA RA RA RA	<4.52 <4.52 <4.36 <4.36 <4.52 <4.52 <4.36 <4.36	<4.52 <4.52 <4.36 <4.36 <4.52 <4.52 <4.36 <4.36
MCP-1	OA OA OA OA RA RA RA RA	1997 1476 >1534 >1534 1463 1083 >1534 >1534	1824 1730 >1534 >1534 2284 1508 >1534 >1534
IL-8	OA OA OA OA RA RA RA RA	28,810 18,797 >7797 >7797 27,905 20,120 >7797 >7797	14,989 18,025 >7797 >7797 24,905 21,055 >7797 >7797
MIP-1α	OA OA OA OA RA RA RA RA	<16.16 <16.16 <1.93 <1.93 <16.16 <16.16 <1.93 <1.93	<16.16 <16.16 <1.93 <1.93 <16.16 <16.16 <1.93 <1.93
IL-10	OA OA OA OA RA RA RA RA	<33.10 <33.10 <32.23 <32.23 <33.10 <33.10 <32.23 <32.23	<33.10 <33.10 <32.23 <32.23 <33.10 <33.10 <32.23 <32.23
G-CSF	OA OA OA OA RA RA RA RA	419.1 3701 5872 36,202 1422 1066 5015 2819	252.9 1328 3586 9641 1231 1155 8282 1270
IL-15	OA OA OA OA RA RA RA RA	21.3 39.1 38.8 50.1 33.0 35.7 57.4 43.6	<20.9 39.3 29.1 29.1 25.1 32.1 52.3 22.8
IL-7	OA OA OA OA RA RA RA RA	<32.96 <32.96 <34.29 <34.29 <32.96 <32.96 <34.29 <34.29	<32.96 <32.96 <34.29 <34.29 <32.96 <32.96 <34.29 <34.29
IL-12(p70)	OA OA OA OA RA RA RA RA	34.2 62.3 <35.41 <35.41 53.2 62.6 59.0 45.0	24.1 59.7 <35.41 <35.41 38.4 63.2 60.6 40.5
IL-17α	OA OA OA OA RA RA RA RA	49.3 64.5 65.7 70.2 57.1 57.3 73.6 56.9	37.1 60.8 47.0 49.4 46.4 57.9 72.4 47.3
IL-9	OA OA OA OA RA RA RA RA	<9.16 <9.16 <8.09 <8.09 <9.16 <9.16 <8.09 <8.09	<9.16 <9.16 <8.09 <8.09 <9.16 <9.16 <8.09 <8.09

**Table 2:** Basal and stimulated levels of cytokines (pg/ml) after a ti-->td:48‐hour-->48-h exposure with TNF-α (10 ng/ml) alone or TNF-α (10 ng/ml) with calcipotriol (10 nM) or dexamethasone (10 nM) or combination of all. The apparent increase of TNF-α was mostly attributable to the added TNF-α. Data comprises cell cultures from 2 to 6 patients with osteoarthritis (OA) or rheumatoid arthritis (RA). The final data is presented in Fig. 9 of the research article (the basal cytokine secretion not included) [1].

**Cytokine**	**Patient**	**no stimulation (basal)**	**TNF-α**	**TNF-α + calcipotriol**	**TNF-α + dex**	**TNF-α+ calcipotriol+ dex**
MIP-1β	OA OA RA RA	<13.77 <13.77 <13.77 <13.77	47.3 128.9 62.8 116.2	34.7 58.9 53.5 85.2		
IL-6	OA OA OA OA OA OA RA RA RA RA RA RA	349.5 301.3 860.9 686.8	>2971 >2971 >2971 >2971 16,350 22,863 7829 4473 5810 21,968 17,409 30,296	1511 2247 2643 2971 4046 3164 3859 2073 3590 9640 7455 10,031	802 1069 774 615 2831 1075 2692 5780	456 397 375 358 1420 585 1387 3568
IFN-γ	OA OA OA OA OA OA RA RA RA RA RA RA	<21.48 <21.48 <21.48 <21.48	41.2 56.7 52.7 38.2 57.4 61.2 67.0 51.0 66.5 88.0 54.4 78.1	26.5 29.4 30.5 33.8 36.5 31.3 40.1 36.0 36.4 55.8 41.6 47.1	24.0 30.7 27.3 29.1 40.8 33.0 36.2 57.5	23.3 24.0 21.8 22.1 31.3 27.6 21.9 41.5
IL-1ra	OA OA RA RA	<88.59 <88.59 <88.59 <88.59	147.1 159.0 148.8 201.0	93.1 <88.6 110.7 127.2		
IL-5	OA OA RA RA	<24.73 <24.73 <24.73 <24.73	<24.73 <24.73 <24.73 <24.73	<24.73 <24.73 <24.73 <24.73		
GM-CSF	OA OA OA OA OA OA RA RA RA RA RA RA	48.19 53.72 51.83 44.88	81.2 77.6 56.3 73.1 70.7 75.7 79.9 88.7 83.4 92.0 63.8 59.7	69.3 67.2 67.4 72.1 50.6 57.1 68.5 67.8 71.2 75.4 58.1 37.3	66.9 68.5 66.5 61.1 70.2 68.2 72.0 76.8	70.8 66.0 61.1 64.1 69.5 70.0 59.7 74.5
TNF-α	OA OA RA RA	<79.83 <79.83 <79.83 <79.83	3160 5322 3421 3062	2298 2077 2533 1598		
RANTES	OA OA OA OA OA OA RA RA RA RA RA	<24.76 <24.76 <24.76 <24.76	606.7 897.8 1267.0 820.6 3973.0 2887.0 302.9 1163.0 862.4 3505 2782	270.5 341.0 447.1 481.2 5040.0 1323.0 180.0 560.1 167.8 3080 2963	339.4 1267.0 660.4 1267.0 371.0 1267.0 672.7	133.6 363.5 268.0 363.8 160.5 442.3 167.9
IL-2	OA OA RA RA	<13.52 <13.52 <13.52 <13.52	<13.52 <13.52 <13.52 15.42	<13.52 <13.52 <13.52 <13.52		
IL-1β	OA OA RA RA	<30.49 <30.49 <30.49 <30.49	<30.49 <30.49 <30.49 <30.49	<30.49 <30.49 <30.49 <30.49		
Eotaxin	OA OA RA RA	<25.51 <25.51 <25.51 <25.51	<25.51 <25.51 <25.51 <25.51	<25.51 <25.51 <25.51 <25.51		
Basic FGF	OA OA OA OA OA OA RA RA RA RA RA RA	<11.22 <11.22 <11.22 <11.22	33.2 30.3 25.5 23.6 23.2 25.9 31.9 27.8 27.0 44.1 24.7 32.5	20.0 24.4 19.6 21.9 19.5 17.5 22.5 22.6 20.3 42.0 20.3 20.8	18.7 22.0 18.0 19.2 22.6 21.05 20.5 28.9	17.9 19.5 17.3 17.8 20.9 19.8 14.3 23.4
VEGF	OA OA OA OA OA OA RA RA RA RA RA RA	142.24 127.62 171.81 164.72	172.7 242.1 211.5 192.3 142.1 175.2 226.5 259.9 186.8 337.7 203.9 182.9	128.0 135.8 120.5 187.9 127.9 117.4 185.0 182.4 132.6 208.6 159.7 201.7	82.3 101.8 91.7 89.8 109.5 132.3 79.4 133.8	69.7 94.2 66.5 77.9 101.8 103.4 65.0 124.8
PDGF-BB	OA OA RA RA	<29.30 <29.30 <29.30 <29.30	<29.30 <29.30 <29.30 <29.30	<29.30 <29.30 <29.30 <29.30		
IP-10	OA OA OA OA OA OA RA RA RA RA RA RA	<33.10 <33.10 <33.10 <33.10	497.2 774.1 771.6 235.0 1761 789.4 275.4 1015 504.5 1727 1040 27,285	100.8 110.8 91.9 73.2 2238 132.7 85.0 258.9 45.8 257.2 475.1 2931	274.3 474.5 230.8 184.6 196.5 518.3 187.4 569.4	146.1 187.5 133.7 105.4 123.8 231.2 62.9 329.8
IL-13	OA OA RA RA	<28.57 <28.57 <28.57 <28.57	<28.57 <28.57 <28.57 <28.57	<28.57 <28.57 <28.57 <28.57		
IL-4	OA OA RA RA	<4.52 <4.52 <4.52 <4.52	<4.52 <4.52 <4.52 <4.52	<4.52 <4.52 <4.52 <4.52		
MCP-1	OA OA RA RA	243.1 305.76 290.81 464.9	1747 2132 1052 824.7	2170 2559 1368 922.6		
IL-8	OA OA RA RA	26.44 26.73 29.68 36.96	11,015 15,848 11,362 9399	7844 8703 6805 6496		
MIP-1α	OA OA RA RA	<16.16 <16.16 <16.16 <16.16	<16.16 <16.16 <16.16 <16.16	<16.16 <16.16 <16.16 <16.16		
IL-10	OA OA RA RA	<33.10 <33.10 <33.10 <33.10	<33.10 <33.10 <33.10 <33.10	<33.10 <33.10 <33.10 <33.10		
G-CSF	OA OA RA RA	<29.78 <29.78 <29.78 <29.78	45.23 55.2 49.75 59.66	<29.78 <29.78 40.69 30.9		
IL-15	OA OA OA OA OA OA RA RA RA RA RA RA	<20.96 <20.96 <20.96 <20.96	40.6 50.9 57.2 42.3 32.4 32.4 44.8 48.3 36.4 51.6 31.4 32.9	25.5 33.2 38.4 22.0 30.3 28.0 30.0 39.4 13.5 44.8 30 31	35.0 48.9 44.6 43.9 43.0 48.9 29.4 51.1	29.9 36.7 34.0 29.8 35.5 40.5 2.9 44.2
IL-7	OA OA RA RA	<32.96 <32.96 <32.96 <32.96	<32.96 <32.96 <32.96 <32.96	<32.96 <32.96 <32.96 <32.96		
IL-12(p70)	OA OA RA RA	<15.08 <15.08 <15.08 20.06	<15.08 <15.08 16.25 15.98	<15.08 <15.08 <15.08 15.57		
IL-17α	OA OA OA OA OA OA RA RA RA RA RA RA	<32.96 <32.96 <32.96 <32.96	34.2 36.1 35.9 30.3 36.8 45.1 40.9 38.0 41.1 55.0 38.3 61.2	<24 25.2 26.0 27.4 28.9 24.9 28.2 28.5 25.6 38.0 30.1 33.0	<24.2 27.1 25.2 <24.2 29.2 30.0 27.4 40.0	<24.2 <24.2 <24.2 <24.2 25.3 26.3 24.3 31.2
IL-9	OA OA RA RA	<9.16 <9.16 <9.16 <9.16	<9.16 <9.16 <9.16 <9.16	<9.16 <9.16 <9.16 <9.16		
PGE2	OA OA OA OA RA RA RA RA		62 100 150 150 150 130 70 280	45 52 60 90 60 60 45 62	35 35 30 32 40 45 38 45	40 31 35 28 55 50 45 38

## 2 Experimental design

Two separate experiments were done: one with IL-1β and 1,25(OH)_2_D_3_ (Table 1) and the other with no stimulus, TNF-α, calcipotriol, dexamethasone and a combination of all stimuli (Table 2). PGE_2_ analysis was done in conjunction with the latter experiment.

### 2.1 Reagents

1,25(OH)_2_D_3_ (Sigma-Aldrich) and calcipotriol (Santa Cruz Biochemistry) were dissolved in 99% ethanol. Recombinant human IL-1β/1F2 (R&D Systems) and dexamethasone (Sigma-Aldrich) were dissolved in sterile PBS and TNF-α (Sigma-Aldrich) in sterile PBS containing 0.1% BSA. 1,25(OH)_2_D_3_ and calcipotriol were stored at −20 °C and cytokines at −80 °C.

### 2.2 Cell culture experiments with inflammatory and anti-inflammatory stimuli

Primary cell cultures were established as previously described [1]. A half of the media was changed two times every week. All analyses were conducted between the third to fifth passages. The synovial stromal cells from six patients with rheumatoid arthritis and six with osteoarthritis were grown in the complete media in four T175 flasks each containing 300,000 cells until they were 70–80% confluent. The flasks were treated either with TNF-α (10 ng/ml), with TNF-α (10 ng/ml) and calcipotriol (10 nM), or with TNF-α (10 ng/ml) and dexamethasone (10 nM) or with a combination of all three compounds for 48 h. Basal secretion of cytokines was analyzed from vehicle (absolute ethanol) treated cells. The synovial stromal cells from four patients with rheumatoid arthritis and osteoarthritis were used for IL-1β/1,25(OH)_2_D_3_ assay. Cells from each patient were cultured in two T175 bottles, one containing IL-1β (0.5 ng/ml) and the other IL-1β (0.5 ng/ml) and 1,25(OH)_2_D_3_ (10 nM) for 48 h. The cell culture media were harvested and stored at −80 °C for the cytokine analysis.

### 2.3 Cytokine analysis

The cytokines present in the medium were quantified with Bio-Rad Bio-Plex Pro Human Cytokine Grp I Panel (27-plex) using the Luminex MagPix Instrument and Luminex xPotent Software.
